# Predictors of caregiver burden among parents of children with neurological impairement

**DOI:** 10.1192/j.eurpsy.2023.2008

**Published:** 2023-07-19

**Authors:** A. Mellouli, S. Zouari, N. Smaoui, O. Jallouli, S. Omri, W. Bouchaala, I. Gassara, S. Ben Nsir, F. Kamoun, M. Maâlej, C. Charfi Triki

**Affiliations:** 1 Psychiatry C Department; 2 Child Neurology Department, Hedi Chaker University Hospital; 3 LR19ES15 laboratory of Child Neurology, Sfax medical school-Sfax university, Sfax, Tunisia

## Abstract

**Introduction:**

Many neurological, sensory and behavioural deficits, are linked with significant limitations in the overall functioning not only of the child but also his/her closest family, and poses a great challenge for the primary parental caregivers.

**Objectives:**

To assess the caregiver burden in parents of children with neurological impairement (NI), and itsrelated factors.

**Methods:**

A total of33 caregivers of children with NI participated in this cross-sectional, descriptive and analytical study, carried out in Child Neurology Department of the University Hospital in Sfax (Tunisia), between February and April 2021.

The Zarit-Caregiver-Burden-Scale (Zarit-CBS) was administered.

**Results:**

The average age of the caregivers (27 mothers and 6 fathers) was 38,33 ± 6,53 years. Among the parents, 17.14% had another disabled child and 30.3% had a mediocre health status. Mother caregivers constitutes the majority of caregiving (82.85%).

The average of the number of children in the family was 1.97±1.18 and the average age of the children (21 boys and 12 girls) was 7,58±4,29 years. Near to the half of them (51,51%) had intellectual disability.Over 54.54% of the children had a functional independence, while 21.21% required help in walking and 24.24% were unable to walk. The intervention was based on motor rehabilitation (57,57%), adequate equipment (24,24%), ergotherapy (45,45%) and speech therapy (60,6%).After the intervention, 63,63% of children had an improvement and 30,3% had a stationary state.

The mean score of Zarit-CBS was 52,45±14,26. The caregiver burden was noted in 96,96%.

The total Zarit-CBS score was associated with the number of children in the family (p=0.047).

There was no significant relationship between Zarit-CBS and the severity of impairement (p=0.418).

**Conclusions:**

Given the variety of factors affecting caregiver burden, specific interventions may promote parental caregivers’well-being, and consequently lead to improved quality of care provided to children with NI.

**Disclosure of Interest:**

None Declared

